# Static and Wind-on Performance of Polymer-Based Pressure-Sensitive Paints Using Platinum and Ruthenium as the Luminophore

**DOI:** 10.3390/s16050595

**Published:** 2016-04-26

**Authors:** Kin Hing Lo, Konstantinos Kontis

**Affiliations:** Division of Aerospace Sciences, School of Engineering, University of Glasgow, University Avenue, Glasgow, G12 8QQ, UK; kostas.kontis@glasgow.ac.uk

**Keywords:** pressure-sensitive paints, contour bumps, supersonic free-stream

## Abstract

An experimental study has been conducted to investigate the static and wind-on performance of two in-house-developed polymer-based pressure-sensitive paints. Platinum tetrakis (pentafluorophenyl) porphyrin and tris-bathophenanthroline ruthenium II are used as the luminophores of these two polymer-based pressure-sensitive paints. The pressure and temperature sensitivity and the photo-degradation rate of these two pressure-sensitive paints have been investigated. In the wind tunnel test, it was observed that the normalised intensity ratio of both polymer-based pressure-sensitive paints being studied decreases with increasing the number of wind tunnel runs. The exact reason that leads to the occurrence of this phenomenon is unclear, but it is deduced that the luminophore is either removed or deactivated by the incoming flow during a wind tunnel test.

## 1. Introduction

Pressure-sensitive paint (PSP) techniques have become a valuable and efficient tool used for surface pressure measurements [1,2,3,4,5,6,7,8]. Comparing to conventional point-based pressure measurement techniques, the advantages of applying pressure-sensitive paint in surface pressure measurements include: (1) pressure information over an entire surface can be obtained; (2) the non-intrusive nature of this experimental technique makes it ideal to be applied on surfaces with any shapes. Furthermore, the flow pattern over the surface of an object can be visualised through the “colour map” obtained from the PSP measurements. Throughout the years, many pressure-sensitive paint formulations have been developed and applied to different scenarios, ranging from typical surface pressure measurements on various objects in wind tunnel tests at different speeds [9,10,11,12,13,14,15] to flow and shock structures visualisation [14,15,16], as well as used for measuring altitudes [17].

**Figure 1:**
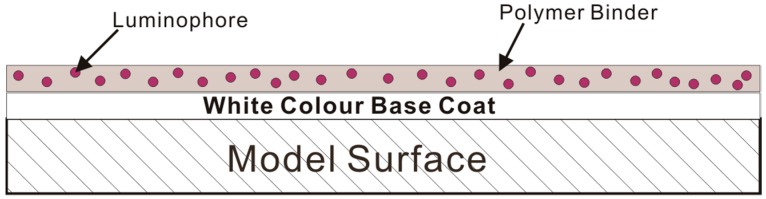
Schematic of the polymer-based pressure-sensitive paint.

The operational principle of polymer-based pressure-sensitive paints has been well documented in the literature, such as Liu and Sullivan [6] and Gregory *et al.* [18]. Therefore, only a brief summary is included here. When a PSP-coated sample is exposed to an excitation light source with the appropriate wavelength, luminophores in the PSP are excited from their ground state (*i.e.*, a lower energy level state) to the excitation state (*i.e.*, a higher energy level state). Since luminophores in the excitation state are unstable, therefore, these unstable luminophores tend to return to the ground state by releasing energy in the forms of light (*i.e.*, luminescent) and heat [6,18,19]. The luminescence emitted by the luminophore molecules has a longer wavelength than its absorption wavelength [6,18]. The difference between the absorption and emission spectra of a luminophore is known as its Stokes shift [20]. The fundamental operational principle of a polymer-based pressure-sensitive paint is by oxygen quenching of the luminophore suspended in an oxygen-permeable binder layer [6,18] (Figure 1). The intensity of luminescence emitted by a luminophore molecule when it returns from the excitation state to its ground state is inversely proportional to the partial pressure of oxygen. To be exact, maximum luminescent emission appears when no oxygen is present (*i.e.*, a vacuum), and the luminescence level decreases with increasing oxygen concentration. Henry’s law states that, at constant temperature, gas pressure at the surface of an object increases linearly with increasing the amount of gas that is dissolved. Through this principle, the luminescence level and the surface pressure of an object can be related.

Response time, pressure sensitivity, temperature sensitivity and the photo-degradation rate are important parameters for characterising the properties of pressure-sensitive paints. Carroll *et al.* [2] concluded that polymer-based pressure-sensitive paints have a typical response time (t_response_) of t_response_ > 0.5 s. Therefore, these pressure-sensitive paints are more suitable for steady surface pressure measurements, such as wind tunnel tests, due to their slow response. Although polymer-based pressure-sensitive paints have been extensively used in various wind tunnel tests, there is lack of available information about the effects of the incoming flow on the performance of the PSPs. Mohsen [21] suggested that the signal intensity of a pressure-sensitive paint changes continuously over time in wind tunnel experiments caused by model movement and deformation. However, it is unclear whether any other factors could affect the signal output from PSPs over time in wind tunnel experiments.

The objective of this study is to investigate the performance change of two in-house-developed polymer-based PSPs in a steady Mach 1.3 free-stream, *i.e.*, M_∞_ = 1.3. A supersonic free-stream is adopted, such that the PSPs being studied are subjected to a stringent test environment. Platinum tetrakis (pentafluorophenyl) porphyrin and tris-bathophenanthroline ruthenium II are used as the luminophores of these two polymer-based PSPs. In addition to the supersonic wind tunnel tests, a series of static calibration experiments are conducted to obtain the pressure sensitivity, temperature sensitivity and photo-degradation characteristics of the paints. It is aimed that the combination of the static and wind-on experimental data can provide a more comprehensive account for the performance of polymer-based PSPs in both static and wind-on conditions.

## 2. Sample Preparation

### 2.1. Aluminium Sheet Sample

Six pieces of Grade 6083 aluminium alloy sheet samples were used in the static calibration experiments. The dimensions of these samples were 40 mm (length) × 40 mm (width) × 3 mm (thickness).

### 2.2. Contour Bump Model

**Figure 2:**
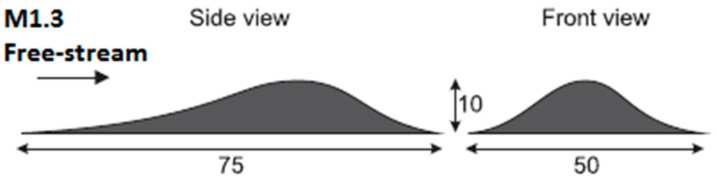
Schematic of the three-dimensional rounded contour bump model.

Two identical three-dimensional rounded contour bump models with the schematic shown in Figure 2 were used in the wind tunnel tests. These models are made of Grade 6083 aluminium alloy. The dimensions of the contour bump models are 75 mm (length) × 50 mm (width) × 10 mm (apex height). The same contour bump model was also employed as a baseline model in various transonic/supersonic wind tunnel tests [15,22,23,24,25]. Therefore, the flow pattern along these contour bump models is well understood, which makes it ideal for the purpose of this study.

### 2.3. Pressure-Sensitive Paints

**Figure 3:**
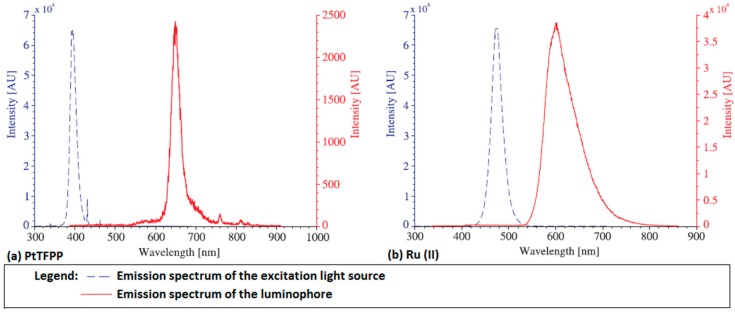
Emission spectra of the: (**a**) platinum tetrakis (pentafluorophenyl) porphyrin (PtTFPP) and (**b**) Ru (II) molecules (red line) and the corresponding emission wavelengths of the illuminating light sources (blue line).

Two in-house-developed polymer-based pressure-sensitive paints were used in this study. These two polymer-based PSPs are a mixture of luminophore powder, ethanol, methyltriethoxysilane (MTEOS) and hydrochloric acid. Platinum tetrakis (pentafluorophenyl) porphyrin (PtTFPP) and tris-bathophenanthroline ruthenium II (Ru (II)) were used as the luminophores. The peak absorption spectra of the PtTFPP and Ru (II) molecules are 395 and 457 nm, respectively [26]. The peak emission spectrum of the PtTFPP molecules is 650 nm when illuminated by an ultra-violet light source with an emission wavelength of 395 nm. Similarly, the peak emission wavelength of the Ru (II) molecules is 560 mm when illuminated by a blue colour light source with an emission wavelength of 475 nm. The emission spectra of the PtTFPP and Ru (II) molecules, as well as the emission wavelengths of the corresponding illumination light sources are shown in Figure 3a,b, respectively.

### 2.4. Sample/Model Preparation Procedures

Although various samples and models were used in this study, the same preparation procedures applied to all of these models and samples. The aluminium sheet samples and contour bump models were first cleaned and degreased by ethanol. Then, the cleaned models/samples were sprayed with five layers of matt white acyclic paint as the base coat. The function of the base coat is to reflect the signal emitted by the luminophore molecules to increase the signal level reaching the CCD (charge couple device) camera [19]. After the base coat was dried, 30 thin layers of PSP were sprayed on the samples/models using an airbrush. The average thickness of the PSP coating was about 15 µm. The PSP-coated samples/models were then cured inside an oven at 70 °C for 7 h. After curing, the PSP-coated samples/models were cooled naturally to the ambient temperature in a dark and dry chamber.

## 3. Experimental Setup

### 3.1. PSP Static Calibration Tests

#### 3.1.1. Pressure and Temperature Sensitivity Tests

**Figure 4:**
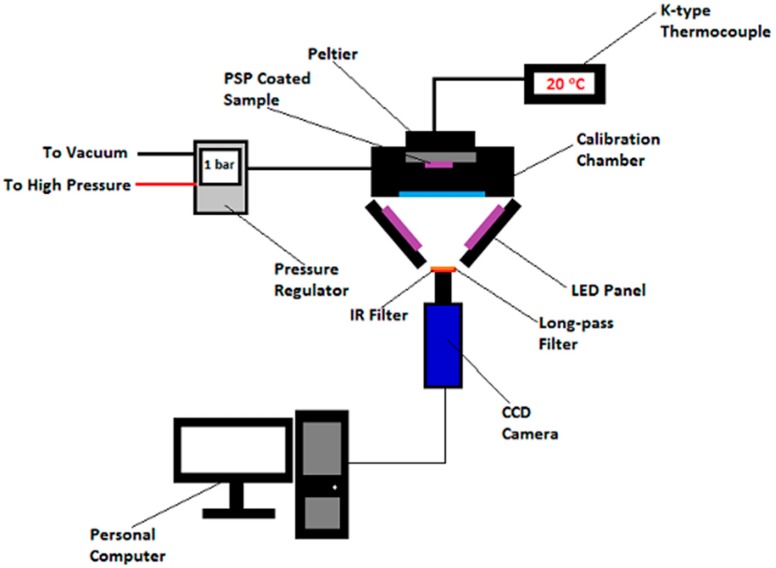
Schematic setup of the pressure-sensitive paint (PSP) calibration experiments.

The pressure and temperature sensitivity of the two polymer-based PSPs used in this study were first calibrated using a sealed static calibration chamber. The calibration chamber has a diameter and depth of 180 and 80 mm, respectively. The PSP-coated samples were placed on the back wall inside the calibration chamber. Optical access to the calibration chamber was achieved via a 5 mm-thick quartz window. Figure 4 shows the schematic setup of the PSP static calibration experiments.

Pressure and temperature within the calibration chamber were monitored and controlled continuously during the calibration tests. A Druck Ltd. DPI 530 pressure controller with an accuracy of approximately 0.001 bar was used to achieve pressure control inside the calibration chamber. Temperature control was handled by a Greenweld Peltier thermo-electric device. The surface temperature of the back wall of the calibration chamber was monitored by a K-type thermocouple. In the pressure sensitivity studies, the absolute pressure inside the calibration chamber was varied progressively from 0 (*i.e.*, vacuum) to 1 bar in a number of step changes. The temperature inside the calibration chamber is maintained at 20 °C throughout for simulating the temperature in the wind tunnel tests. In contrast, in the temperature sensitivity studies, the temperature inside the calibration chamber was varied progressively from 20 to 60 °C, while the absolute pressure was maintained constant at 1 bar. It should be noted that after each step of temperature change, a five minute settling time was provided before any data were taken in order to ensure the equilibrium condition was achieved within the calibration chamber.

Light emitting diode (LED) panels were used to provide uniform illumination in the PSP calibration experiments. A pair of ultra-violet (UV) LED panels with a maximum emission wavelength of 395 nm was used to illuminate the PtTFPP polymer-based PSP-coated sample. Similarly, illumination of the Ru (II) polymer-based PSP-coated sample was achieved through a pair of 475-nm blue LED panels. Each of these LED panels contains 225 individual LEDs arranged in a 15 × 15 array. The illuminating light source was switched off during the pressure and temperature adjustments in order to minimise the photo-degradation effect to the PSPs. Signals emitted from the PSP-coated samples were captured by a LaVision Image Intense 12-bit CCD camera, which was controlled by the software Davis 7.2. The frame rate and the exposure time of the camera were set to 8 frames per second and 50 ms, respectively. A 540-nm long-pass filter and an infra-red cut-off filter were placed in front of the camera lenses to separate the luminescence emitted by the PSPs from the excitation light source and heat signals.

Thirty “dark images” (*i.e.*, the images captured without any illumination) were captured and summed prior to each set of the PSP calibration experiment. In the PSP calibration tests, for each pressure and temperature, thirty “bright” images (*i.e.*, the images captured with illumination) were captured and summed. Summation of the images can improve the signal-to-noise ratio (SNR) and, therefore, increases the accuracy of the result obtained. The “dark corrected images” were obtained by subtracting the summed “bright images” with the summed “dark image”. This process aims to minimise the effect caused by background noise [19]. This “dark corrected images” were then stored in a Windows-based personal computer and processed by an in-house developed MATLAB program.

#### 3.1.2. Pressure-Sensitive Paint Photo-Degradation Studies

The experimental setup of the PSP photo-degradation studies is very similar to the PSP calibration experiments described in the previous Section 3.1.1. However, throughout the entire period of the photo-degradation test, the pressure and temperatures inside the calibration chamber were maintained at 1 bar and 20 °C, respectively.

### 3.2. PSP Winds Tunnel Tests

In the wind tunnel experiments for investigating the wind-on performance of the two polymer-based PSPs being studied, an intermittent in-draft transonic wind tunnel was used. The same wind tunnel was also employed in a wide range of supersonic flow related studies [15,23,24,25,27,28,29,30,31,32,33]. The test section of the wind tunnel has dimensions of 485.5 mm (length) × 150 mm (width) × 216.5 mm (height). Optical access was achieved through the two quartz-made side windows and the ceiling mounted top window. The required Mach 1.3 supersonic free-stream was obtained by expanding the airflow from the inlet through a pair of supersonic nozzles situated upstream of the test section. The wind tunnel has a stable runtime of 6 s. At M_∞_ = 1.3, the flow Reynolds number per unit length (Re/L) is 11.7 × 10^6^ [25]. Under the same initial conditions, the free-stream Mach number variation in the test section is M_∞_ = 1.3 ± 0.01 [24].

**Figure 5:**
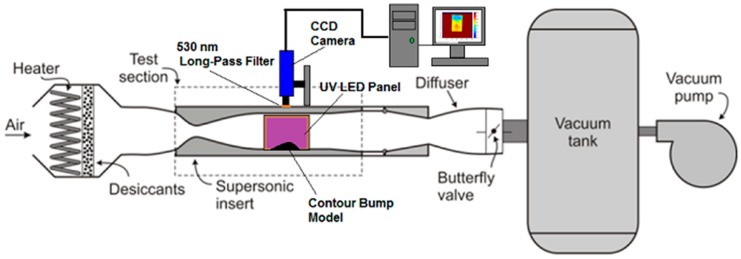
Schematic setup of the PSP wind tunnel (dynamic) experiments.

A schematic setup of the PSP wind tunnel tests is shown in Figure 5. During the tests, the PSP-coated contour bump model was floor mounted in the wind tunnel test section. Illumination of the PSP-coated models was achieved using a pair of (i) UV LED panels for the PtTFPP polymer-based PSP-coated model or (ii) blue LED panels for the Ru (II) polymer-based PSP-coated model. The same pairs of LED panels were used in the PSP static calibration tests. The illuminating light source was placed at the two sides of the wind tunnel test section, so that the entire test section was uniformly illuminated. In order to minimise the photo-degradation effect, the excitation light source was only switched on during the period of each wind tunnel test, *i.e.*, 5 s for capturing wind-on images plus another 5 s for capturing wind-off images immediately after the wind tunnel operation. Complete darkness was maintained within the laboratory during any other periods of time.

Signals emitted from the PSP-coated model during the wind tunnel tests were captured by a LaVision Image Intense 12-bit CCD camera, which was also used in the PSP static calibration experiments. The frame rate and the exposure time of the camera were set to 8 frames per second and 50 ms, respectively. Similar to the PSP static calibration tests, thirty “dark images” were captured and summed immediately prior to the start of each wind tunnel test for conducting the dark correction process. In total, forty “wind-on” images were captured during each wind tunnel test (*i.e.*, 5 s) followed by capturing another forty “wind-off” images immediately after the wind tunnel operation. Wind-off images were captured after each wind tunnel test in order to account for the temperature effects caused by shock and expansion waves to the contour bump model [26,34]. Captured images were stored in a Windows-based computer and post-processed by an in-house-developed MATLAB program. It should be noted that the first 10 “wind-on” and “wind-off” images captured in each wind tunnel test were removed from further processing in order to account for the unsteadiness effects during the wind tunnel start-and-stop period [25]. As a result, thirty pairs of “wind-on” and “wind-off” images captured were summed separately for calculating the contour of the signal intensity ratio of the contour bump model.

In total, twenty-four sets of wind tunnel tests were conducted per PSP-coated contour bump model. A five-minute interval was provided between two consecutive wind tunnel runs for vacuuming the vacuum tank of the wind tunnel. It should be noted that the temperature within the wind tunnel test section was monitored continuously by two k-type thermocouples. These two thermocouples were situated at 25 mm upstream and 15 mm downstream of the front and rear ends of the contour bump model, respectively. The average temperature fluctuation (∆T_∞_) during wind-off condition over the entire period of wind tunnel test was about 20 ± 0.5 °C. Since the PSP-coated contour bump model was exposed to the excitation light source for 10 seconds during each wind tunnel test, therefore, the overall light exposure time of each PSP-coated model after the twenty-fourth wind tunnel experiment was 240 s.

## 4. Results and Discussion

### 4.1. Pressure Sensitivity

**Figure 6:**
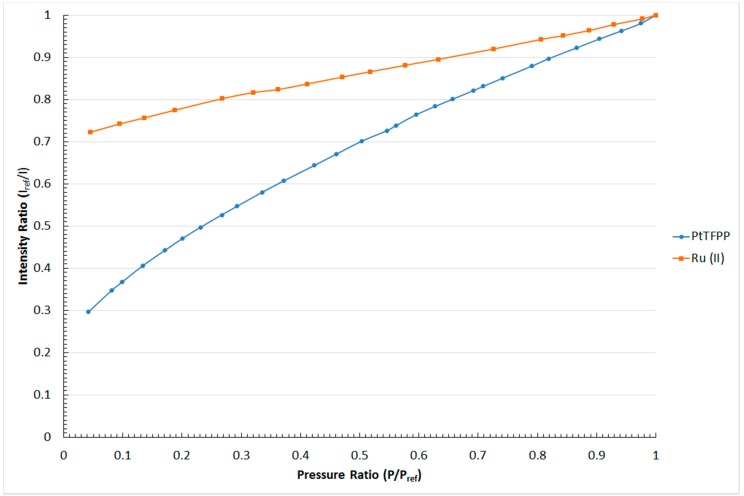
Stern–Volmer plot for the PtTFPP and Ru (II) polymer-based pressure-sensitive paints.

The pressure sensitivity of the PtTFPP and Ru (II) polymer-based PSPs is shown in the form of Stern–Volmer plot in Figure 6. A Stern–Volmer plot relates the normalised intensity output (I_ref_/I) to the normalised pressure ratio (P/P_ref_) of a pressure-sensitive paint. It should be noted that the reference pressure used in this study was 1 bar. From Figure 6, it can be seen that considerably linear Stern–Volmer relations exist in both polymer-based PSPs being studied. To be exact, the relation between I_ref_/I and P/P_ref_ in these two polymer-based PSPs is considerably linear. A linear fit model was used to determine the gradient of the Stern–Volmer relation, which represents the pressure sensitivity of a PSP. The linear fit Stern–Volmer relations obtained for the two polymer-based PSPs being studied are tabulated in Table 1.

As shown in Table 1, the pressure sensitivity of the PtTFPP and Ru (II) polymer-based pressure-sensitive paints is 0.7% and 0.27% per unit pressure change, respectively. This indicates that the PSP using PtTFPP as the luminophore shows higher pressure sensitivity than the paint with Ru (II) as the luminophore. It can be observed from Figure 6 that a much steeper gradient is shown in the Stern–Volmer plot of the PtTFPP rather than the Ru (II) polymer-based PSP. With this result, it is able to be concluded that the PtTFPP polymer-based PSP performs better in pressure measurements that involve small levels of pressure changes.

### 4.2. Temperature Sensitivity

**Figure 7:**
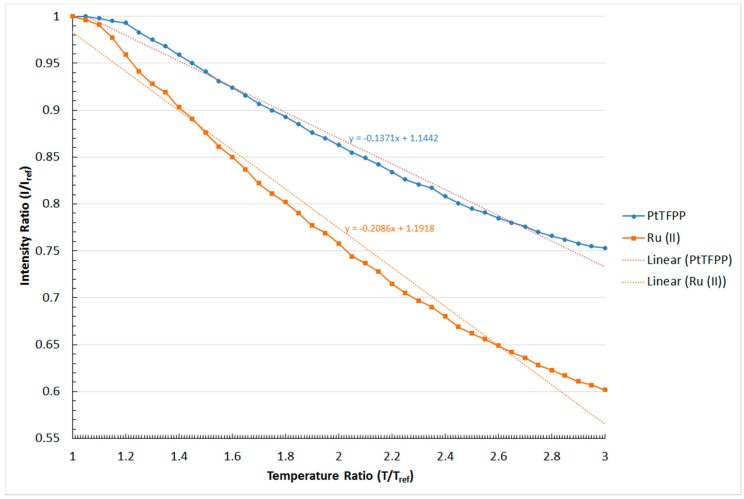
Temperature sensitivity of the PtTFPP and Ru (II) polymer-based PSPs.

One of the major drawbacks in pressure measurements using pressure-sensitive paints is their temperature dependency [6,18,26]. Therefore, it is necessary to investigate the temperature sensitivity characteristics of the two polymer-based PSPs being studied. Figure 7 shows the relation between the normalised intensity output (I_ref_/I) and temperature ratio (T/T_ref_) of the PtTFPP and Ru (II) polymer-based PSPs used in this study. It should be noted that the reference temperature (T_ref_) used was 20 °C.

The result shown in Figure 7 indicates that the normalised intensity output decreases in a relatively linear manner with increasing temperature ratio for both the PtTFPP and Ru (II) polymer-based PSPs. This is because the rate of oxygen quenching is proportional to the activation energy of the oxygen molecules. To be exact, the rate of oxygen quenching increases, and hence, the normalised intensity output decreases when the polymer-based PSPs are subjected to higher temperatures. Relatively small changes in terms of the normalised intensity ratio are observed in both the PtTFPP and Ru (II) polymer-based PSPs when the temperature ratio is approaching T/T_ref_ = 1. In fact, a similar phenomenon was also documented in the studies conducted by Yang *et al.* [13] and Gongora-Orozco *et al.* [35]. However, the authors in [13] and [35] did not explain the reasons that might lead to the occurrence of this phenomenon. Similar to the pressure sensitivity, the gradient of the plotted lines in Figure 7 shows the temperature sensitivity of the two polymer-based PSPs being studied. From Figure 7, it can be seen that the change in the normalised intensity output per unit temperature ratio increment for the PtTFPP and Ru (II) polymer-based PSPs is −0.014 and −0.021, respectively. Therefore, the signal intensity outputted by the polymer-based PSP using Ru (II) as the luminophore is more susceptible to temperature changes than the PtTFPP polymer-based PSP.

### 4.3. Photo-Degradation

**Figure 8:**
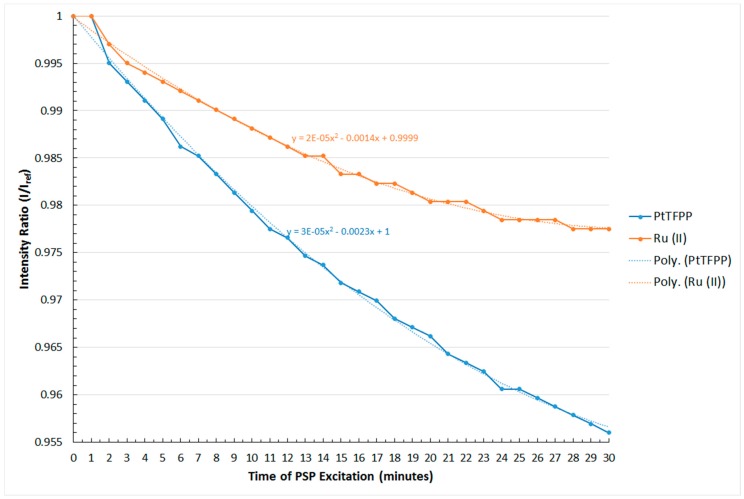
Photo-degradation rate of the PtTFPP and Ru (II) polymer-based PSPs.

In pressure-sensitive paints, the output signal intensity decreases with increasing exposure time to the excitation light source [6,18]. This is known as the photo-degradation effect of PSPs. The photo-degradation rate of PSPs could be varied from 1% [36] to 47% [37] per hour depending on the substrate, luminophore and the formulation used. Therefore, this effect could significantly affect the accuracy of surface pressure measurements using PSPs. As a result, the photo-degradation rate of the two polymer-based PSPs being studied is investigated, and the result obtained is shown in Figure 8.

In general, the signal outputted from both the PtTFPP and Ru (II) polymer-based PSPs decreases with increasing excitation time as seen in Figure 8. Although a second-order polynomial fit relation is adopted here, it can be seen from Figure 8 that the non-linear term in the equations for both the PtTFPP and Ru (II) polymer-based PSPs used is negligible compared to the linear term. Therefore, the photo-degradation rate of these two polymer-based PSPs can be obtained by calculating the gradient of the plotted lines in Figure 8. After calculation, it can be seen that the photo-degradation rate of the PtTFPP and Ru (II) polymer-based PSPs are −0.14% and −0.07% per minute, respectively. This means that the decay rate of the signal intensity output of the PtTFPP polymer-based PSP is twice that of the Ru (II) polymer-based PSP. The result obtained indicates that the PtTFPP polymer-based PSP is less stable than the Ru (II) polymer-based PSP when exposed to the excitation light source.

### 4.4. Behaviour of Polymer-Based PSPs in Wind Tunnel Tests

After considering the pressure sensitivity, temperature sensitivity and photo-degradation characteristics of the PtTFPP and Ru (II) polymer-based PSPs being studied, their behaviour in the supersonic wind tunnel tests is now investigated. As two identical three-dimensional rounded contour bumps were used in these wind tunnel experiments, therefore, the typical flow pattern over the bump model in a Mach 1.3 supersonic free-stream is first shown before discussing the performance of the two polymer-based PSP being studied in wind tunnel tests.

#### 4.4.1. Flow Pattern over the Three-Dimensional Rounded Contour Bump

**Figure 9:**
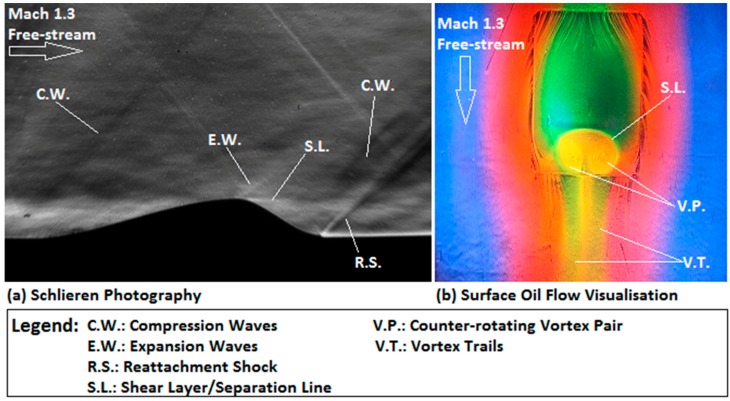
Flow pattern over the three-dimensional rounded contour bump. (**a**) Schlieren photography image and (**b**) surface oil flow visualisation image.

Figure 9 shows the streamwise and spanwise flow patterns over the three-dimensional rounded contour bump in a Mach 1.3 free-stream in the form of the Schlieren and surface oil flow visualisation images. The setups of the Schlieren photography and surface oil flow visualisation experiments are identical to those employed in [24,25], and therefore, the details are not included here.

The streamwise flow pattern (Figure 9a) along the contour bump can be summarised as follows. The front end of the rounded contour bump deflects the incoming flow, so that a series of compression waves (C.W.) are formed at the beginning of the bump. Across these compression waves, the flow moves along the ramp-shape front surface of the bump to reach the bump crest. Flow expansion begins at the bump crest through a series of expansion waves (E.W.). Due to the rapid change in the bump geometry, flow separation appears downstream of the bump crest. This leads to the formation of a low pressure wake region in the bump valley, which is evidenced by the presence of the shear layer (S.L.) downstream of the bump crest. Finally, a reattachment shock (R.S.) is formed at the rear end of the bump across which the flow becomes parallel to the free-stream direction. For the spanwise flow pattern, it can be seen from Figure 9b that the incoming flow is deflected at the beginning of the bump to move along and around the bump. A separation line (S.L.) is shown at the bump crest, which indicates the location where flow separation begins. The presence of the low pressure wake region downstream of the bump crest attracts the flow from the two sides of the bump to move into and circulates in the bump valley. As a result, a pair of counter-rotating vortices (V.P.) is formed in the bump valley. These vortices propagate downstream, and therefore, a pair of vortex trails (V.T.) is left behind the rear end of the bump model.

To conclude from the flow pattern as seen in Figure 9, it is expected that relatively high pressure regions exist in the ramp-shaped front surface of the bump due to the flow compression effect caused by the compression waves. In contrast, the presence of the wake region and the spanwise vortices in the bump valley indicates that a low pressure zone exists downstream of the bump crest. With this information, the capability of the PtTFPP and Ru (II) polymer-based PSPs being studied in resolving the surface pressure profile of the contour bump model can be discussed.

#### 4.4.2. Surface Pressure Map

**Figure 10:**
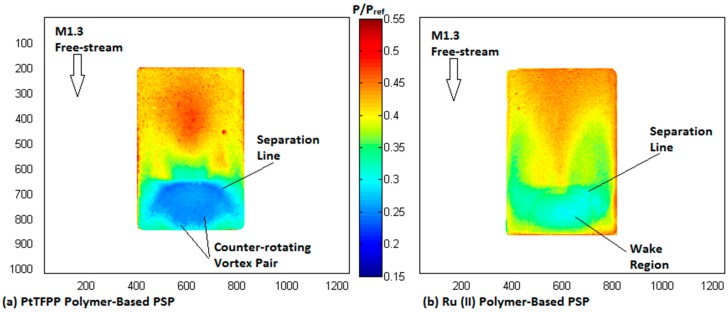
Surface pressure map of the contour bump measured by: (**a**) PtTFPP and (**b**) Ru (II) polymer pressure-sensitive paints.

The surface pressure contour of the rounded contour bump measured by using the PtTFPP and Ru (II) polymer-based pressure-sensitive paints is shown in Figure 10. It should be noted that during the photo-registration process, the intensity ratio contour over the surface of the contour bump model was obtained by dividing the summed wind-on images with the summed wind-off image captured immediately after the wind tunnel test. Therefore, the thermal effect that acted on the model during the wind tunnel test was compensated. In addition, although no data of pressure measurements using pressure taps and transducers are provided here, the data shown in [38] using a double-ramp model concluded that accurate surface pressure measurements in supersonic free-stream could be achieved by using the two polymer-based PSPs employed in the present study, as the same polymer-based PSP formulations were also used in [38].

In the surface pressure map corresponding to the PtTFPP polymer-based PSP (Figure 10a), relatively high pressure regions are shown in the entire front surface of the bump upstream of the bump crest. The presence of the three-dimensional flow relieving effects weaken the flow compression at the two sides of the bump; therefore, a zone that shows the highest pressure ratio exists at the centre portion of the bump front surface. Downstream of the bump crest, as expected, low pressure zones appear in the entire bump valley due to the presence of the wake region there. In addition, the separation line at the bump crest and the spanwise vortices in the bump valley are able to be resolved by the PtTFPP polymer-based PSP. This indicates that the PtTFPP polymer-based PSP can provide both the qualitative surface pressure map and quantitative information about the flow pattern over the contour bump model. It should be noted that a similar contour map pattern was also documented in [38] using a contour bump model coated with polymer-based PSP with PtTFPP as the luminophore.

In contrast, a comparably poor resolution surface pressure map as shown in Figure 10b is obtained with the Ru (II) polymer-based PSP-coated contour bump model. Similar to the case when the PtTFPP polymer-based PSP is used, relatively high pressure regions are shown in the front surface of the bump. However, the Ru (II) polymer-based PSP failed to resolve the highest pressure zone at the centre portion of the bump front surface. Downstream of the bump crest, it can be seen from Figure 10b that relatively low pressure regions exist in the bump valley. However, the Ru (II) polymer-based PSP can neither resolve the details of the spanwise vortex pair in the bump valley nor the separation line at the bump crest. Therefore, compared to the PtTFPP polymer-based PSP, the Ru (II) polymer-based PSP shows limited capability in providing qualitative information about the flow features that present in the flow field. Similar conclusion was also drawn by Zare-Behtash *et al.* [38] using a contour bump model coated with Ru (II) polymer-based PSP. It should be noted that the PSP formulation used in [38] is slightly different from the one employed in this study.

**Figure 11:**
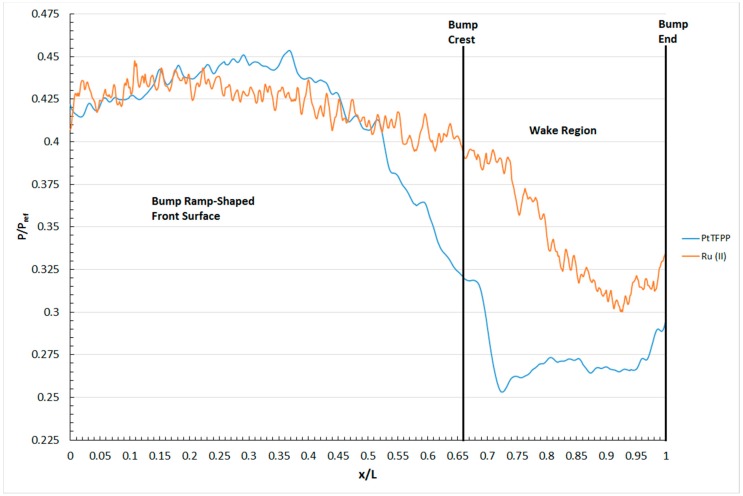
Surface pressure profile along the centreline of the contour bump measured by the two polymer-based pressure-sensitive paints.

The lack of resolution in the surface pressure contour generated by the Ru (II) polymer-based PSP is due to its poorer pressure sensitivity compared to the PtTFPP polymer-based PSP. To be exact, the Ru (II) polymer-based PSP shows limited capability in resolving small pressure variations in the flow field. Instead, it tends to “averaging” the surface pressure distribution over the surface of an object. This is evidenced by the absence of the flow features, like the spanwise vortex pair and the separation line in the surface pressure map shown in Figure 10b. Similar conclusions were also drawn by Zare-Behtash *et al.* [38] and Quinn *et al.* [26,34]. In fact, an interesting phenomenon can be observed by plotting the surface pressure profile, measured by the two PSPs being studied, along the centreline of the contour bump, as shown in Figure 11. From Figure 11, it can be seen that near the front end of the bump (*i.e.*, 0 < x/L<0.25), the surface pressure profile measured by the two polymer-based PSPs is very similar. However, when the normalised distance is increased to x/L > 0.2, the surface pressure profiles measured by the two PSPs start to deviate from each other. Significant deviation can be observed when the normalised distance is increased to x/L = 0.25 and beyond, for which the surface pressure level measured by the PtTFPP polymer-based PSP is considerably lower than that measured by the Ru (II) polymer-based PSP. It is deduced that the difference in the pressure sensitivity between these two polymer-based PSPs caused various pressure levels being measured in the bump valley of the contour bump. In fact, similar conclusions were also drawn by Quinn *et al.* [26,34] and Zare-Behtash *et al.* [38] when comparing the centreline pressure profiles obtained using PtTFPP and Ru (II) polymer-based pressure-sensitive paints in subsonic and hypersonic free-stream, respectively.

#### 4.4.3. Mechanical Degradation of Pressure-Sensitive Paints

**Figure 12:**
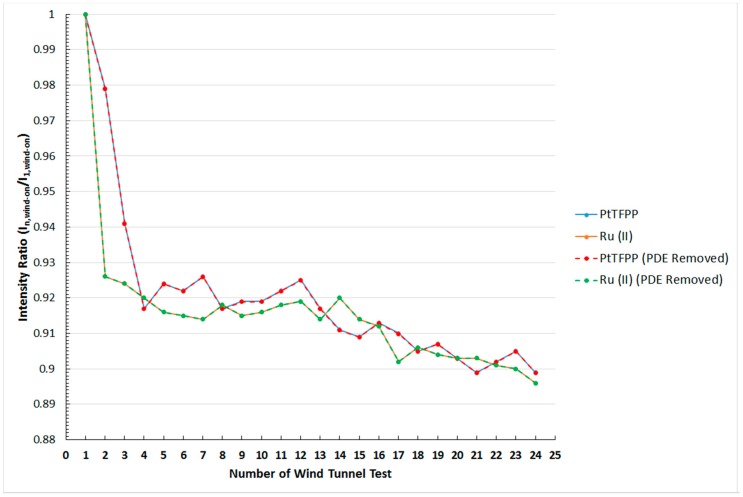
Relation between the normalized intensity ratio and the number of wind tunnel test for the PtTFPP and Ru (II) polymer-based pressure-sensitive paints.

The variation of the normalised intensity output with time of the PtTFPP and Ru (II) polymer-based PSPs in the wind tunnel tests is shown in Figure 12. It should be noted that the intensity ratio is obtained by dividing the signal intensity outputted by the PSP during wind-on condition in each individual wind tunnel test with that of the first wind tunnel test (*i.e.*, I_n,wind-on_/I_1,wind-on_). In general, it can be seen from Figure 12 that the signal intensity emitted by both the PtTFPP and Ru (II) polymer-based PSPs decreases with increasing the number of wind tunnel runs. To be exact, the output signal intensity of both paints drops continuously over time. Since the PSP-coated model was exposed to the excitation light source during the period of wind tunnel operation, the corresponding photo-degradation effect caused by the model should be first removed in order to investigate the actual effects caused by the supersonic free-stream to the PSP-coated model. The results obtained after removing the photo-degradation effect are presented in the form of dashed lines in Figure 12 with the red and green lines representing the PtTFPP and Ru (II) polymer-based PSPs, respectively.

In fact, it can be observed from Figure 12 that the effect caused by photo-degradation on the two PSPs being studied is not significant as the lines that represent the result obtained with and without photo-degradation corrections coincide. This is expected due to the short exposure time of the PSP-coated model to the excitation light source during the wind tunnel tests. In terms of the signal output, the normalised intensity ratio (I/I_1_) of the PtTFPP and Ru (II) polymer-based PSPs obtained after the twenty-fourth wind tunnel experiment are 0.899 (blue solid line) and 0.896 (orange solid line), respectively. The result obtained implies that both the PtTFPP and Ru (II) polymer-based PSPs being studied overestimate the surface pressure of the model by approximately 10% after twenty-four wind tunnel tests in a Mach 1.3 supersonic free-stream.

In terms of the temperature effects, the contour bump model was subjected to the same test conditions during the wind tunnel experiments. Therefore, the model experienced the same thermal effects caused by shock waves and expansion waves during each wind tunnel test. As a result, the change in the output intensity caused by the temperature effects is eliminated when the intensity output of the PSPs during wind-on condition measured at each individual wind tunnel test is normalised with that measured at the first wind tunnel test (*i.e.*, I_n,wind-on_/I_1,wind-on_). Of course, this argument is only valid when the surface temperature of the contour bump remained considerably constant throughout the entire period of the experiment. Egami *et al.* [39] noticed that models made of aluminium sprayed with white colour base coat and PSP show lower thermal conductivity than uncoated models. However, the temperature measured by the two thermocouples located slightly upstream and downstream of the contour bump model did not show any significant temperature change throughout the entire period of the wind tunnel test. In addition, by comparing the wind-off intensity ratio of the PSP obtained between each individual wind tunnel test and the first wind tunnel test (*i.e.*, I_n,wind-off_/I_1,wind-off_), it was observed that this ratio was extremely close to unity for both PSPs being studied. From this, it is suggested that the surface temperature of the contour bump models remains considerably constant throughout the entire period of the wind tunnel tests.

It is believed that the exposure of the PSP-coated model to the strong incoming flow causes the output signal intensity of both the PtTFPP and Ru (II) polymer-based PSPs to drop continuously over time. To be exact, it is deduced that the strong incoming flow either removed or deactivated some luminophores in the PSPs during the wind tunnel experiments. From this, it is deduced that a new type of degradation effect called the “mechanical degradation” effect exists. Unfortunately, the hypothesis about the presence of the “mechanical degradation” effect could not be proven in this study. This is because the variation of the paint thickness with the number of wind tunnel test could not be measured as the surface profiler available in the author’s institution does not have a sufficiently large test section to accommodate the contour bump model. However, Basu and Kamble [40] mentioned that polymer-based PSPs using triethoxysilane (TEOS) based sol-gel show a tendency to form cracks and peel-off from the model surface.

It should be noted the data gathered from the present study did not show any signs of paint removal. However, the phenomenon of PSP peel-off from the model surface does happen based on the authors’ actual observation form both subsonic, supersonic and hypersonic wind tunnel tests. This phenomenon usually happened when a model is sprayed with a polymer-based PSP with a high sol-gel to catalyst/solvent ratio. Here, it must be emphasised that using a polymer-based PSP with a high sol-gel to solvent/catalyst ratio is sufficient, but does not necessarily means cracks and/or loose paint layers must be developed. In fact, based on the authors’ experienced that even though the same preparation procedures and test conditions are implemented, the probability of seeing cracks and/or loose layers in the models sprayed with polymer-based PSPs with a high sol-gel to solvent/catalyst ratio is around 50%. Therefore, it seems like some unknown additional factors actually contribute to this crack and/or loose paint layer formation process. Further investigations are required in order to fully understand the chemical property of TEOS-based pressure-sensitive paints.

In addition, the drop in the signal intensity outputted might also be caused by luminophore deactivation. In fact, this phenomenon appears occasionally in polymer-based PSPs using PtTFPP and Ru (II) as luminophores, as reported by Amao [41]. There is one point here that should be noted based on actual wind tunnel test experience. In general, it was observed from a range of wind tunnel experiments with wind speeds ranging from subsonic to hypersonic using various models that progressive whitening of the pressure-sensitive paint appears during the experiments. Moreover, it was observed that the colour change to the PSP-coated models is reversible, and the colour of the model tends to return to original after a period of idle time. The duration of the idle time required depends on the formulation used. Therefore, it is required to investigate further about the chemical properties of TEOS-based pressure-sensitive paint in order to understand what causes the PSP model whitening and the “mechanical degradation” phenomena to occur.

Therefore, on top of the corrections to the temperature and photo-degradation effects, corrections to the decay in the output signal intensity of polymer-based PSPs over time are also required in wind tunnel experiments. This is particularly important in experiments that require being repeated multiple times or that last for a long period of time. In terms of the performance of the two PSPs being studied in the wind tunnel tests, for the PtTFPP polymer-based PSP, it can be seen from Figure 12 that the output signal intensity drops abruptly in the first four consecutive wind tunnel runs. To be exact, the normalised intensity ratio dropped from I_1_/I_1_ = 1 after the first wind tunnel test to I_4_/I_1_ = 0.917 after the fourth wind tunnel experiment. This indicates that the output signal intensity is reduced by 8.3% after four consecutive wind tunnel tests. Afterwards, the reduction in the output signal intensity becomes gradual, and the average change in the output signal intensity of the PtTFPP polymer-based PSP between the fourth and twenty-fourth wind tunnel tests is about −1.96%. A similar trend could also be observed when the Ru (II) polymer-based PSP is used. From Figure 12, it can be seen that a 7.4% drop in the signal output intensity appears after the first two consecutive wind tunnel tests when Ru (II) polymer-based PSP is used. Changes in the output signal intensity of the Ru (II) polymer-based PSP become gradual after the second wind tunnel run. The average change in the normalised intensity ratio per wind tunnel experiment is about −0.14% between the second and the twenty-fourth wind tunnel tests.

The reason that leads to the abrupt reduction in the output signal intensity of the PtTFPP and Ru (II) polymer-based PSPs in the first several consecutive wind tunnel tests is also unclear. It is deduced that the first several layers of PSP coating on the model surface are relatively loose. These loose layers of paint are prone to be removed by the strong incoming flow during the wind-on condition. This might explain why the normalised intensity ratio drops rapidly over the first several wind tunnel runs in both the PtTFPP and Ru (II) polymer-based PSPs used in this study. In addition, compared to the Ru (II) polymer-based PSP, the PtTFPP polymer-based PSP is more suitable to be used in cases when the PSP-coated model is only tested in the wind tunnel for a limited number of times. This is because the output signal intensity reduction is less susceptible in the PtTFPP polymer-based PSP in the first couple of consecutive wind tunnel tests. However, comparable performance is observed in both the PtTFPP and Ru (II) polymer-based PSPs being studied when the PSP-sprayed model is required to be used repeatedly over a long period of time.

## 5. Conclusions

An experimental study has been conducted to investigate the static and wind-on performance of two polymer-based pressure-sensitive paints using PLATINUM TETRAKIS (pentafluorophenyl) porphyrin (*i.e.*, PtTFPP) and tris-bathophenanthroline ruthenium II (*i.e.*, Ru (II)) as the luminophores. The static calibration tests were conducted in a sealed calibration chamber, and the wind-on experiments were conducted in a transonic wind tunnel in a Mach 1.3 supersonic free-stream. In the static calibration tests, it was observed that the pressure sensitivity of the PtTFPP polymer-based pressure-sensitive paint is higher than that of the Ru (II) polymer-based pressure-sensitive paint. It was concluded that the PtTFPP polymer-based pressure-sensitive paint shows better performance in measuring small pressure variations in a flow field due to its higher pressure sensitivity. In addition, it was observed that the polymer-based PSP using PtTFPP as the luminophore is less prone to temperature variations than the Ru (II) polymer-based pressure-sensitive paint. However, it was found that the PtTFPP polymer-based PSP shows a two-times higher photo-degradation rate than the Ru (II) polymer-based PSP.

In the wind tunnel tests, a new type of degradation called the “mechanical degradation” was proposed. It was emphasised that when a polymer-based PSP-coated model is used in the wind tunnel tests, on top of the corrections for the temperature sensitivity and photo-degradation effects, the mechanical degradation effect must also be corrected. It was observed that for both the PtTFPP and Ru (II) polymer-based PSPs, the output signal intensity of the PSP-coated model decreases continuously over time. For both the PtTFPP and Ru (II) polymer-based PSPs being studied, approximately a 10% reduction in the output signal intensity was observed after twenty-four consecutive wind tunnel experiments. This corresponds to an over-estimation of the surface pressure ratio of the model by about 10%. The presence of the “mechanical degradation effect” was deduced to be caused by the strong incoming flow either by deactivating or removing some luminophores from the PSP-coated model during the wind tunnel experiments. Moreover, it has been seen that for both the PtTFPP and Ru (II) polymer-based pressure-sensitive paints, the output signal intensity of the paints dropped abruptly over the first several consecutive wind tunnel experiments. The underlying reason that causes this phenomenon is currently unclear. It was deduced that the top several layers of the pressure-sensitive paint on a model surface are loose, so that they can easily be removed by the incoming flow during the first several consecutive wind-on experiments.

Finally, it was mentioned that the PtTFPP polymer-based pressure-sensitive paint is more suitable than the Ru (II) polymer-based pressure-sensitive paint to be used in experiments that only last for a very short duration or only require being repeated a limited number of times. In contrast, both the PtTFPP and Ru (II) polymer-based PSPs are suitable for those experiments that need to be repeated many times or have a long test duration, because they show similar long-term performance.

## Figures and Tables

**Table 1:** Pressure-sensitive of the PtTFPP and Ru (II) polymer-based PSP.

Type of PSP	Linear Fit Stern–Volmer Relation
PtTFPP Polymer	$\frac{I_{ref}}{I}=0.3252+0.7029\frac{P}{P_{ref}}$
Ru (II) Polymer	$\frac{I_{ref}}{I}=0.7203+0.2777\frac{P}{P_{ref}}$
